# Viral Zoonoses in Small Wild Mammals and Detection of Hantavirus, Spain

**DOI:** 10.3201/eid2806.212508

**Published:** 2022-06

**Authors:** Silvia Herrero-Cófreces, François Mougeot, Tarja Sironen, Hermann Meyer, Ruth Rodríguez-Pastor, Juan José Luque-Larena

**Affiliations:** Universidad de Valladolid, Palencia, Spain (S. Herrero-Cófreces, R. Rodríguez-Pastor, J.J. Luque-Larena);; Instituto de Investigación en Recursos Cinegéticos, Ciudad Real, Spain (F. Mougeot);; University of Helsinki, Helsinki, Finland (T. Sironen);; Bundeswehr Institute of Microbiology, Munich, Germany (H. Meyer)

**Keywords:** hantavirus, *Apodemus sylvaticus*, arenavirus, zoonoses, *Microtus arvalis*, *Mus spretus*, poxvirus, rodent-borne viruses, serologic screening, viruses, Spain, vector-borne infections

## Abstract

We screened 526 wild small mammals for zoonotic viruses in northwest Spain and found hantavirus in common voles (*Microtus arvalis*) (1.5%) and high prevalence (48%) of orthopoxvirus among western Mediterranean mice (*Mus spretus*). We also detected arenavirus among small mammals. These findings suggest novel risks for viral transmission in the region.

Wildlife viromes harbor potentially threatening zoonoses for humans that require increased effort in identification and surveillance (*1*). Rodents are considered main reservoirs of emerging zoonoses (*2*), and the large population fluctuations of reservoir species play a key role in modulating infection risk (*3*). Anthropogenic land-use changes, agricultural intensification, and irrigation also favor rodent invasions and risk for pathogen spillover (*4*). The common vole (*Microtus arvalis*) is a widespread rodent inhabiting intensified farming landscapes in northwestern Spain, where population numbers and pathogen prevalence lead to spillover of zoonotic bacteria such as *Francisella tularensis* and *Bartonella* spp. (*5*).

We report the prevalence of rodent-borne zoonotic viruses in Europe (i.e., hantavirus, arenavirus [lymphocytic choriomeningitis virus (LCMV)], and orthopoxvirus) (*6*) among the small mammals inhabiting farming landscapes. We also report the effect of natural fluctuations of common vole numbers on viral prevalence (phase dependence). Our study was conducted in intensively farmed landscapes, in the Tierra de Campos region of Castilla-y-León, northwestern Spain (*7*), where the small mammal population is mainly composed of 4 species: common vole, long-tailed field mouse (*Apodemus sylvaticus*), western Mediterranean mouse (*Mus spretus*), and greater white-toothed shrew (*Crocidura russula*) (*7*). 

We live-trapped small mammals during March 2013–March 2019. We collected samples from blood, spleen, liver, and lungs by using standard protocols and stored them at –23°C until molecular analysis could be performed (Appendix). We owned all necessary licenses and permits for conducting this study. 

We detected specific hantavirus, LCMV, and orthopoxvirus IgG in serum samples by using immunofluorescence assay. We used fluorescein isothiocyanate (FITC) anti-IgG as a secondary antibody and evaluated all slides under a fluorescence microscope. For molecular analysis, we isolated RNA from liver and lung tissues and DNA from a mix of liver and spleen. We performed single-step reverse transcription PCR (RT-PCR) for LCMV detection in the liver, nested reverse transcription PCR for hantavirus detection in lung samples, and conventional pan-poxvirus PCR method followed by an additional orthopoxvirus-specific PCR for orthopoxvirus detection in the mix samples. We used generalized linear models to test variations of prevalence between species and calculate prevalence in common voles according to host sex (male or female), trapping month (March, July, or November), and population density phase (increase, peak, or crash). 

We screened 526 individual animals from 4 species for the presence of 3 viruses (Table; Appendix). We found evidence of hantavirus infection only in common voles, at an average prevalence of 1.6% (95% CI 0.6%–3.3%; 7/438). Positive results for LCMV infection (either by immunofluorescence assay or PCR) were detected in 5.9% (95% CI 0.7%–19.7%) of long-tailed field mice (2/34, 11.1% (95% CI 0.7%–48.2%) of shrews (1/9), and 2.2% (95% CI 1.1%–4.0%) of common voles (10/458). Orthopoxvirus IgG was present in 1.3% (95% CI 0.4%–3.0%) of common voles (5/382) and in 48% (95% CI 27.8%–68.7%) of western Mediterranean mice (12/25), and we observed significant differences between both species (χ^2^ = 59.643, d.f. = 3; p < 0.001). In long-tailed field mice, we only detected LCMV during summer (July). In common voles, we found no effect of cycle phase or month on virus prevalence (Appendix), but LCMV prevalence differed between sexes (χ^2^ = 5.189, d.f. = 1; p = 0.023) and was higher in males (3.7%; 95% CI 1.6%–7.1%) than in females (0.8%; 95% CI 0.1%–0.3%).

**Table:** Prevalence of hantavirus, arenavirus (LCMV), and orthopoxvirus in 4 small mammal species from the Tierra de Campos region, Castilla-y-León, northwest Spain, 2013–2019*

Species	Common name	Virus	Prevalence		
			Screening method	No. positive/screened	% Positive (95% CI)
*Apodemus sylvaticus *	Long-tailed field mouse	LCMV	IFA	2/34	5.9 (0.7–19.7)
			PCR	0/2	Not tested
		Hantavirus	IFA	0/34	Not tested
			PCR	Not tested	Not tested
		Orthopoxvirus	IFA	0/34	Not tested
			PCR	Not tested	Not tested
*Crocidura russula *	Greater white-toothed shrew	LCMV	IFA	0/7	Not tested
			PCR	1/9	11.1 (0.3–48.2)
		Hantavirus	IFA	0/7	Not tested
			PCR	0/9	Not tested
		Orthopoxvirus	IFA	0/7	Not tested
			PCR	Not tested	Not tested
*Microtus arvalis *	Common vole	LCMV	IFA	8/382	2.1 (0.9–4.1)
			PCR	2/89	2.2 (0.3–7.9)
		Hantavirus	IFA	3/382	0.8 (0.2–2.3)
			PCR	4/62	6.5 (1.8–15.7)
		Orthopoxvirus	IFA	5/382	1.3 (0.4–3.0)
			PCR	0/243	Not tested
*Mus spretus *	Western Mediterranean mouse	LCMV	IFA	0/25	Not tested
			PCR	Not tested	Not tested
		Hantavirus	IFA	0/25	Not tested
			PCR	Not tested	Not tested
		Orthopoxvirus	IFA	12/25	48.0 (27.8–68.7)
			PCR	Not tested	Not tested
All hosts		LCMV	All tests	13/526	2.5 (1.3–4.2)
		Hantavirus	All tests	7/458	1.5 (0.6–3.1)
		Orthopoxvirus	All tests	17/510	3.3 (2.0–5.3)

*LCMV, lymphocytic choriomeningitis virus.

Recent surveys of viral zoonoses in Spain have shown low antibody prevalence of LCMV (1.7%) (*8*) and hantavirus (0.06%) (*9*) among humans. Hantavirus antibodies were detected in red foxes (*Vulpes vulpes*) (*10*), and LCMV antibodies were detected in long-tailed field mice and red foxes (*8*,*10*). Our study detected hantavirus in a wild rodent reservoir in Spain. The reported prevalence was low (1.6%) and did not differ between the phases of the common vole population cycle. However, the cyclic dynamic of this rodent host, which harbored all 3 virus species screened, may influence the risks associated with contact with infected rodents. Common voles can reach densities of up to 1,000 per hectare during population peaks, so the infected proportion may become a considerable public health concern. Orthopoxvirus infection risk is of growing concern in Europe because of the absence of smallpox vaccination among the human population <45 years of age (*6*). Because half of all the western Mediterranean mice analyzed were positive for orthopoxvirus, the potential transmission risk for the virus from this rodent to humans should be considered and further confirmed with larger sample sizes.

Further investigation is required regarding the molecular nature and infectivity of the hantavirus and orthopoxvirus detected, as well as their circulation pathways, which will help to uncover possible transmission routes and determine more precisely the level of infection risk to human populations. Our results can be used by local authorities to refine virus surveillance, including clinical diagnosis of new viruses, and improve public health strategies to prevent and minimize zoonotic risks for persons living in areas recurrently affected by outbreaks linked to common voles.

Appendix Additional information about viral zoonoses in small wild mammals and detection of hantavirus, Spain.
