# Supplementary material for: Viral Zoonoses in Small Wild Mammals and Detection of Hantavirus, Spain
Source: Emerg Infect Dis. 2022 Jun;28(6):1294–6. doi: 10.3201/eid2806.212508 (PMC9155867; doi:10.3201/eid2806.212508)
Supplement: Appendix — Additional information about viral zoonoses in small wild mammals and detection of hantavirus, Spain. [file 21-2508-Techapp-s1.pdf]

Article DOI: https://doi.org/10.3201/eid2806.212508

Viral Zoonoses in Small Wild Mammals and Detection of Hantavirus, Spain

Appendix

Study Site and Small Mammal Guild

The study was conducted in the "Tierra de Campos" region, Castilla-y-León, NW Spain, in farming landscapes dominated by cereal fields with scattered irrigated crops (mainly alfalfa) and interspersed by fallows and remnant semi-natural vegetation (*1*). Climate is continental-Mediterranean, characterized by wide seasonal temperature oscillations: long winters with frequent frost events, hot summers with drought periods, and precipitation concentrated during spring and autumn (*2*).

In the studied habitats, more than 95% of the small mammal community belongs to a guild composed of three rodents and one insectivore: common voles (*Microtus arvalis*), long-tailed field mice (*Apodemus sylvaticus*), western Mediterranean mice (*Mus spretus*), and great white-toothed shrews (*Crocidura russula*) (*1*). The common vole is a fossorial rodent (*3*) characterized by population peaks recorded every 3 years in the study area (*4*), whereas mouse species show seasonal fluctuations (*5*,*6*). The great white-toothed shrew is the main insectivore species in the guild (*7*).

Trapping Design and Sample Collection

Fieldwork consisted of periodic live-trapping events three times per year (March, July, November) from March 2013 to March 2019. Our sampling design was spatially stratified, monitoring randomly selected fields from those available among the three most relevant crops (i.e., cereals, alfalfa and fallows). We used Sherman© traps (8 cm × 9 cm × 23 cm; LFAHD Sherman©) baited with carrot and apple, set them open in the morning and checked them 24 h later. See (*1*) and (*4*) for more details. Each trapped animal was provided with a unique code; date, site and crop field were recorded.

Once in the laboratory, animals were euthanized with CO_2, following a humane protocol approved by the ethics committee of our institution (CEEBA, Universidad de Valladolid; authorization code: 4801646). Immediately after death, animals were weighed, sexed and blood was collected by cardiac puncture using a 25G needle with a sterile 1.5 mL syringe and coated with heparin. Samples were centrifuged at 3000 rpm for 20 min. Serum was collected and stored at −23°C. Animals trapped from 2013 to 2018 were stored at −23°C until dissection; however, animals trapped in 2019, were dissected immediately following euthanasia. Following standard protocols, the spleen, liver, and lungs were individually weighted (±0.005 g) and stored at −23°C until molecular analysis could be performed, except for animals trapped in 2019, which were frozen in Invitrogen® RNAlater® Stabilization Solution (Thermo Fisher Scientific Inc., Waltham, MA, USA). We screened 526 individuals from four species of the small mammal guild for the presence of three viruses (https://wwwnc.cdc.gov/EID/article/28/6/21-2508-T1.htm; Appendix Tables 1 and 2).

Viruses Screened: Hantavirus, Arenavirus and Orthopoxvirus

Hantaviruses are RNA rodent-borne viruses belonging to the family Bunyaviridae transmitted by aerosols from rodent excreta. They cause persistent infection in hosts. In Europe, there are five different hantaviruses: Tula virus, carried by *M. arvalis*; Puumala virus, by bank voles (*Myodes glareolus*); Dobrava virus, by yellow-necked field mice (*A. flavicollis*); Saarema virus, by striped field mouse (*A. agrarius*); and Seoul virus, by rats (*Rattus norvegicus*, *R. rattus*) (*8*,*9*). Arenaviruses are RNA viruses belonging to the family Arenaviridae, possibly transmitted by rodent fluids. They cause chronic infection in hosts. Lymphocytic choriomeningitis virus (LCMV) is the only arenavirus endemic in Europe. The house mouse (*M. musculus*) is the main reservoir and carrier of this virus although it can circulate in other rodent species (*8*,*10*). Orthopoxviruses are DNA viruses that can be directly transmitted by rodents and other hosts such as cats. Cowpox virus is the main virus of this type in Europe and causes a rapidly recovering infection in hosts. Some voles (*Myodes* spp., *Microtus* spp.) and mice (*Apodemus* spp.) are the main reservoirs (*8*,*11*).

Serologic Screening

Specific IgG antibodies were detected from specimens with serum samples available using an IFA following Forbes et al. (*12*). Hantavirus-, LCMV-, or orthopoxvirus-infected Vero cells mixed with uninfected cells were spotted and fixed on the IFA slide. The slides were stored at −70 ◦C until use.

The samples were diluted 1:20 in phosphate-buffered saline solution (PBS) and tested for hantavirus virus, LCMV, and orthopoxvirus. Seropositive human serum was used as a positive control for hantavirus and orthopoxvirus-IFA tests, while positive mouse serum was the positive control for LCMV-IFA. PBS was used as a negative control in all cases. Slides were warmed up to room temperature and 25 µL of each diluted sample, positive and negative controls were added in independent wells. The slides were incubated at +37°C in a moist chamber for 30 min, followed by three washing steps in PBS for 5 min each and one last washing step with Milli-Q water. After slides were dried under a fun, a 25 µL amount of secondary antibody was used in every well. Fluorescein (FITC) AffiniPure Fab Fragment Goat Anti-Mouse IgG (H+L) (Jackson InmunoResearch, Ely, UK), diluted 1:30, was used as secondary antibody in animal sample, negative control and LCMV positive control wells. Fluorescein (FITC) AffiniPure $F(ab')_2$ Fragment Goat Anti-Human IgG (H+L) (Jackson InmunoResearch, Ely, UK), diluted 1:100, was used in hantavirus and orthopoxvirus positive control wells. After incubation at +37°C in a moist chamber for 30 min, slides were washed three times in PBS for 5 min each time and one last washing step with Milli-Q water. When completely dry, coverslips were placed on the slides with a mounting medium, and slides were evaluated under a fluorescence microscope. Slides were protected from light and stored at +4°C. Samples with unclear results were repeated, diluting the original sample 1:10, 1:40 and 1:80.

Molecular Detection

Small mammals with positive results to hantavirus or LCMV, and those with undetermined results (i.e., common voles with no serum available and all the shrews), were selected for PCR analyses focused on RNA viruses' detection. RNA was isolated by using Invitrogen® TRIzol® Reagent (Thermo Fisher Scientific Inc., Waltham, MA, USA), following

the manufacturer's instructions. RNA concentration was measured with a Nanodrop® ND-1000 spectrophotometer.

For LCMV, a total of 100 liver samples (10 IFA-positives, 5 common voles with unclear IFA results, 76 common voles with no sera available and 9 shrews) were tested. One-step reverse transcription PCR (RT-PCR) was performed for arenavirus detection, using Old-World Arena Rivigene primers and Invitrogen® one-step RT-PCR kit (Thermo Fisher Scientific Inc., Waltham, MA, USA). RT-PCR reaction was performed using Thermal cycler. The cycling protocol consisted of six steps: reverse transcription step 30 min at 50°C; followed by one temp step of 2 min at 94°C; 45 cycles of 20 s at 94°C; one 30-second step at 55°C; one 60-second step at 72°C; and a final step of 5 min at 72°C. In each PCR run, positive and negative controls were included. Finally, PCR products were run on agarose gel and visualized under UV light. See (*13*) for more details.

For hantavirus, we checked 71 lung samples (3 IFA-positives, 3 common voles with unclear IFA result, 56 common voles with no sera available and 9 shrews). RT reaction mixture was prepared using RevertAid Premium Reverse transcription (Thermo Fisher Scientific Inc., Waltham, MA, USA) and a previous phase of RNA amplification was performed as follows: 30 min at 55°C and 5 min at 85°C. Nested RT-PCR was done for hantavirus detection, using Phusion Flash High-Fidelity PCR Master Mix (Thermo Fisher Scientific Inc., Waltham, MA, USA). The first PCR to obtain cDNA was carried out as follows: an initial step of 10 s at 98°C; 7 cycles of 1 s at 98°C, followed by 5 s at 50°C and 15 s at 72°C; 35 cycles of 1 s at 98°C, followed by 5 s at 55°C and 15 s at 72°C; and a last step of 1 min at 72°C. The second PCR to obtain DNA comprised an initial step of 10 s at 98°C; 40 cycles of 1 s at 98°C, followed by 5 s at 60°C and 15 s at 72°C; and a last step of 1 min at 72°C. The second round PCR products were visualized in agarose gels and visualized under UV light. See (*14*) for more details.

A total of 243 common voles sampled from March 2013 to March 2015 (62 animals with no sera available) were tested for orthopoxvirus DNA (using a mix of liver and spleen). DNA was isolated with QIAamp DNA Mini Kit® (QIAGEN, Valencia, CA, USA) and measured with Nanodrop ND-1000. The pan-poxvirus PCR method (*15*) and a real-time PCR using the RealStar® Orthopoxvirus PCR Kit 1.0 (Altona Diagnostics, Hamburg, Germany) were used. This kit includes three independent PCRs: (i) an internal control to detect and avoid problems

with inhibition, (ii) a PCR that specifically detects variola virus and (iii) an OPV-specific PCR, respectively (*16*). The reaction mix was set up according to the manufacturer's instructions.

Statistical Analysis

We obtained information on the prevalence of each host species. Prevalence data were summarized as the proportion of infected hosts (the number of infected hosts among hosts examined) and 95% confidence intervals (CI). We used generalized linear models (GLM) to test variations of prevalence between species, and prevalence in host species according to host sex (male, female), trapping month (March, July, November) and population density phase (increase, peak, crash) (Appendix Table 1). Because of sample size limitations, the prevalence model was calculated only for common voles (Appendix Table 2). A p-value of <0.05 was considered significant. We used the "lme4" package (*17*) of the R software version 3.6.1 (https://cran.r-project.org/bin/windows/base/old/3.6.1).

References

1. Rodríguez-Pastor R, Luque-Larena JJ, Lambin X, Mougeot F. "Living on the edge": the role of field margins for common vole (*Microtus arvalis*) populations in recently colonised Mediterranean farmland. Agric Ecosyst Environ. 2016;231:206–17. https://doi.org/10.1016/j.agee.2016.06.041

2. Rivas-Martínez S, Penas Á, del Río S, Díaz González TE, Rivas-Sáenz S. Bioclimatology of the Iberian Peninsula and the Balearic Islands. In: Loidi J, editor. The vegetation of the Iberian Peninsula. Cham (Switzerland): Springer International Publishing; 2017. p. 29–80.

3. Hernández MC, Sánchez-González B, Navarro-Castilla Á, Barja I. Topillo campesino—*Microtus arvalis* (Pallas, 1779). Enciclopedia virtual de los vertebrados españoles. 2017 Mar 14 [cited 2021 Oct 20]. https://digital.csic.es/bitstream/10261/146730/4/micarv_v1.pdf

4. Mougeot F, Lambin X, Rodríguez-Pastor R, Romairone J, Luque-Larena JJ. Numerical response of a mammalian specialist predator to multiple prey dynamics in Mediterranean farmlands. Ecology. 2019;100:e02776. PubMed https://doi.org/10.1002/ecy.2776

5. Torre I, Arrizabalaga A, Díaz M. Ratón de campo (*Apodemus sylvaticus*. Linnaeus, 1758). Galemys. 2002;14:1–26.

6. Palomo LJ, Justo ER, Vargas JM. *Mus spretus* (Rodentia: Muridae). Mamm Species. 2009;840:1–10. https://doi.org/10.1644/840.1

7. Palomo LJ, Gisbert J, Blanco JC. Atlas y libro rojo de los mamíferos terrestres de España. Madrid: Dirección General para la Biodiversidad; 2007. p. 588.

8. Kallio-Kokko H, Uzcategui N, Vapalahti O, Vaheri A. Viral zoonoses in Europe. FEMS Microbiol Rev. 2005;29:1051–77. PubMed https://doi.org/10.1016/j.femsre.2005.04.012

9. Vapalahti O, Mustonen J, Lundkvist A, Henttonen H, Plyusnin A, Vaheri A. Hantavirus infections in Europe. Lancet Infect Dis. 2003;3:653–61. PubMed https://doi.org/10.1016/S1473-3099(03)00774-6

10. Meerburg BG, Singleton GR, Kijlstra A. Rodent-borne diseases and their risks for public health. Crit Rev Microbiol. 2009;35:221–70. PubMed https://doi.org/10.1080/10408410902989837

11. Kinnunen PM, Henttonen H, Hoffmann B, Kallio ER, Korthase C, Laakkonen J, et al. Orthopox virus infections in Eurasian wild rodents. Vector Borne Zoonotic Dis. 2011;11:1133–40. PubMed https://doi.org/10.1089/vbz.2010.0170

12. Forbes KM, Voutilainen L, Jääskeläinen A, Sironen T, Kinnunen PM, Stuart P, et al. Serological survey of rodent-borne viruses in Finnish field voles. Vector Borne Zoonotic Dis. 2014;14:278–83. PubMed https://doi.org/10.1089/vbz.2013.1526

13. Vieth S, Drosten C, Lenz O, Vincent M, Omilabu S, Hass M, et al. RT-PCR assay for detection of Lassa virus and related Old World arenaviruses targeting the L gene. Trans R Soc Trop Med Hyg. 2007;101:1253–64. PubMed https://doi.org/10.1016/j.trstmh.2005.03.018

14. Klempa B, Fichet-Calvet E, Lecompte E, Auste B, Aniskin V, Meisel H, et al. Hantavirus in African wood mouse, Guinea. Emerg Infect Dis. 2006;12:838–40. PubMed https://doi.org/10.3201/eid1205.051487

15. Li Y, Meyer H, Zhao H, Damon IK. GC content-based pan-pox universal PCR assays for poxvirus detection. J Clin Microbiol. 2010;48:268–76. PubMed https://doi.org/10.1128/JCM.01697-09

16. Olson VA, Laue T, Laker MT, Babkin IV, Drosten C, Shchelkunov SN, et al. Real-time PCR system for detection of orthopoxviruses and simultaneous identification of smallpox virus. J Clin Microbiol. 2004;42:1940–6. PubMed https://doi.org/10.1128/JCM.42.5.1940-1946.2004

17. Bates D, Mächler M, Bolker B, Walker S. Fitting linear mixed-effects models using lme4. J Stat Softw. 2015;67.

Appendix Table 1. Number of samples analyzed from each trapping session in the "Tierra de Campos" region, Castilla-y-León, NW Spain, 2013–2019

Year	Month	Phase	Host*(sample size)				
			AS	CR	MA	MS	All species
2013	All months	Increase	10	7	51	10	78
	March		0	1	4	0	5
	July		5	6	15	0	26
	November		5	0	32	10	47
2014	All months	Peak	8	2	183	10	203
	March		3	1	63	6	73
	July		2	0	101	1	104
	November		3	1	19	3	26
2015	All months	Crash	2	0	15	0	17
	March		2	0	8	0	10
	July		0	0	0	0	0
	November		0	0	7	0	7
2016	All months	Peak	0	0	63	0	63
	March		0	0	3	0	3
	July		0	0	30	0	30
	November		0	0	30	0	30
2017	All months	Crash	0	0	61	0	61
	March		0	0	30	0	30
	July		0	0	30	0	30
	November		0	0	1	0	1
2018	All months	Increase	0	0	4	0	4
	March		0	0	1	0	1
	July		0	0	3	0	3
	November		0	0	0	0	0
2019	All months	Peak	14	0	81	5	100
	March		14	0	81	5	100
All years	All months		34	9	458	25	526

*AS, *Apodemus sylvaticus*; CR, *Crocidura russula*; MA, *Microtus arvalis*; MS, *Mus spretus*

Appendix Table 2. Results of generalized linear models (GLM) to test variations of prevalence in *Microtus arvalis* from "Tierra de Campos" region, Castilla-y-León, NW Spain, 2013–2019

Predictor	LCVM	Hantavirus	Orthopoxvirus
Sex	X^2 = 5189; df = 1, p = 0023	X^2 = 0204; df = 1, p = 0651	X^2 = 0292; df = 1, p = 0589
Month	X^2 = 3354; df = 2, p = 0187	X^2 = 0046; df = 2, p = 0977	X^2 = 1265; df = 2, p = 0531
Phase	X^2 = 4200; df = 2, p = 0122	X^2 = 2054; df = 2, p = 0358	X^2 = 2449; df = 2, p = 0294
